# Seamless Insert-Plasmid Assembly at High Efficiency and Low Cost

**DOI:** 10.1371/journal.pone.0153158

**Published:** 2016-04-13

**Authors:** Roger M. Benoit, Christian Ostermeier, Martin Geiser, Julia Su Zhou Li, Hans Widmer, Manfred Auer

**Affiliations:** 1 Laboratory of Biomolecular Research, Paul Scherrer Institut, Villigen, Switzerland; 2 Novartis Pharma AG, Basel, Switzerland; 3 Novartis Institutes for BioMedical Research, Basel, Switzerland; 4 The Kellog School of Science and Technology, The Scripps Research Institute, La Jolla, United States of America; 5 University of Edinburgh, School of Biological Sciences (CSE) and School of Biomedical Sciences (CMVM), Edinburgh, United Kingdom; Tulane University Health Sciences Center, UNITED STATES

## Abstract

Seamless cloning methods, such as co-transformation cloning, sequence- and ligation-independent cloning (SLIC) or the Gibson assembly, are essential tools for the precise construction of plasmids. The efficiency of co-transformation cloning is however low and the Gibson assembly reagents are expensive. With the aim to improve the robustness of seamless cloning experiments while keeping costs low, we examined the importance of complementary single-stranded DNA ends for co-transformation cloning and the influence of single-stranded gaps in circular plasmids on SLIC cloning efficiency. Most importantly, our data show that single-stranded gaps in double-stranded plasmids, which occur in typical SLIC protocols, can drastically decrease the efficiency at which the DNA transforms competent *E*. *coli* bacteria. Accordingly, filling-in of single-stranded gaps using DNA polymerase resulted in increased transformation efficiency. Ligation of the remaining nicks did not lead to a further increase in transformation efficiency. These findings demonstrate that highly efficient insert-plasmid assembly can be achieved by using only T5 exonuclease and Phusion DNA polymerase, without *Taq* DNA ligase from the original Gibson protocol, which significantly reduces the cost of the reactions. We successfully used this modified Gibson assembly protocol with two short insert-plasmid overlap regions, each counting only 15 nucleotides.

## Introduction

**Fig 1:**
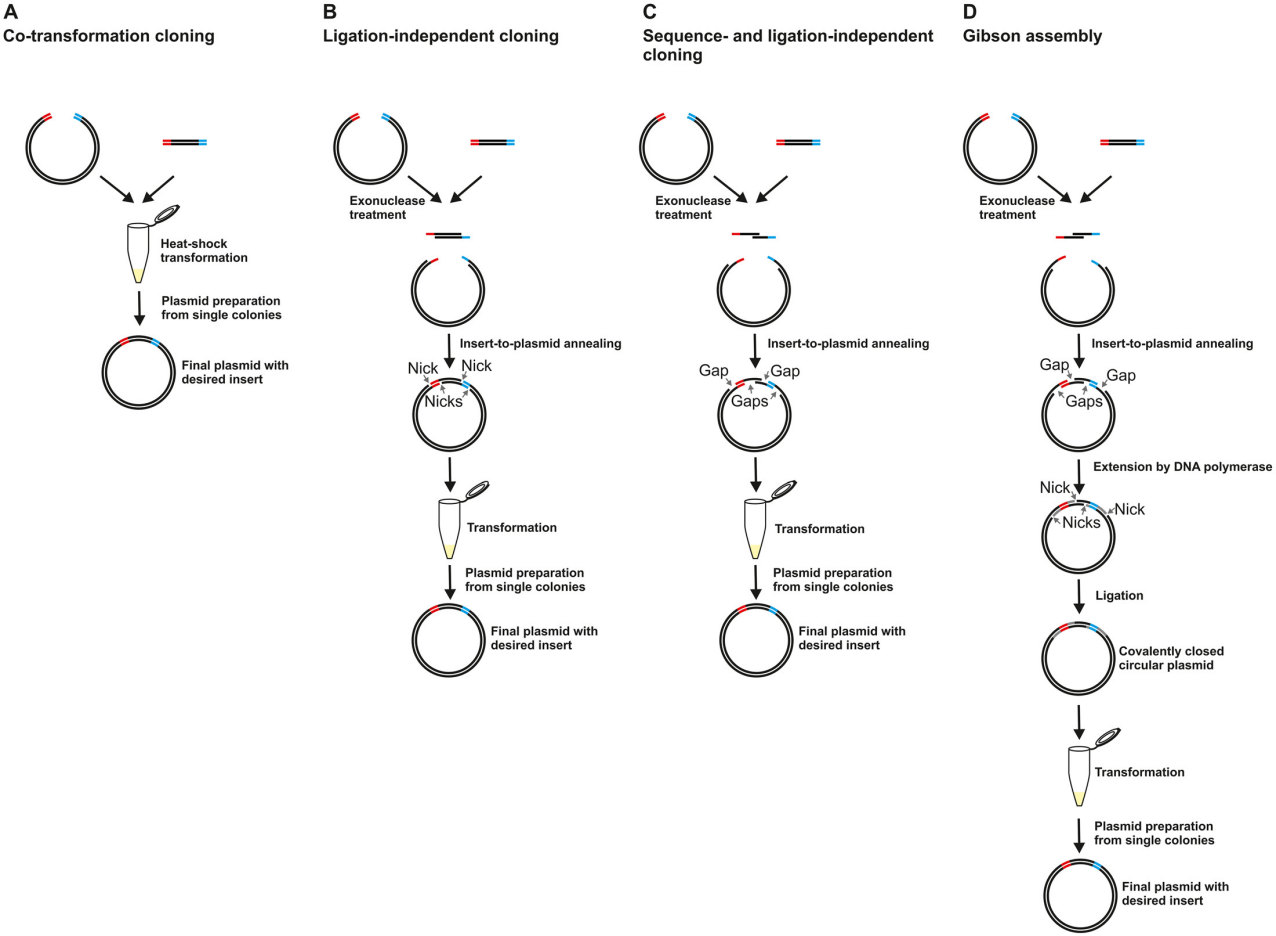
Methods overview. A) Co-transformation cloning [2, 3]. The two homology regions are shown in red and blue. B) Ligation-independent cloning (LIC) [6] using complementary single-stranded ends of defined length. C) Sequence- and ligation-independent cloning (SLIC) [7, 8] using single-stranded ends of undefined length. After insert-to-plasmid annealing, single-stranded regions of excessive length result in single-stranded gaps (small arrows) in the circularized plasmids. D) Gibson assembly [9]. After insert-to-plasmid annealing at the overhangs of undefined length, the 3’-ends serve as primers for extension by DNA polymerase, which fills in the single-stranded gaps. Finally, the nicks are ligated.

Linear DNA fragments can be seamlessly and directionally inserted into linearized plasmids by simply co-transforming chemically competent *E*. *coli* cells with the insert-plasmid mixture [1–4]. Insert and linear plasmid assemble at required ~12–20 nucleotides long terminal (or non-terminal) homology regions through an *E*. *coli* repair or recombination event that is not fully understood, but works independently of *Rec*A in strains typically used for molecular cloning [5] (Fig 1A).

Although much simpler, the basic principle is comparable to strategies used in genome editing, such as for example the currently very popular CRISPR-Cas9 technology [10]: When a double-stranded break is introduced into a DNA molecule (chromosome or plasmid), the cells’ endogenous homology-directed repair machinery can at this site introduce any DNA fragment that is flanked by matching homology regions. While direct co-transformation of *E*. *coli* cells with two linear DNA fragments (insert and plasmid) is unsurpassed in simplicity amongst seamless cloning methods, the low cloning efficiency and the requirement for ultracompetent chemically competent *E*. *coli* cells for reliable cloning can limit the usefulness of this approach. Interestingly, electroporation is not a suitable transformation method for co-transformation cloning using standard *E*. *coli* strains [2, 11].

The cloning efficiency can be drastically increased by rendering the terminal homology regions of both, insert and plasmid, single-stranded, to allow *in vitro* insert-to-plasmid annealing at complementary overhangs (ligation-independent cloning, LIC) [6]. Many LIC protocols use specific strategies to produce single-stranded overhangs of precisely defined length [6, 12–14]. Insert-to-plasmid annealing at single-strands that have exactly the length of the complementary sequences results in circularly closed, double-stranded DNA molecules with nicks, but no gaps (Fig 1B). Such plasmids can directly, without ligation, be used to transform competent *E*. *coli* cells. Inside the cells, the nicks are probably ligated and the plasmid replicates. A disadvantage of such LIC methods is that they impose sequence restrictions at the complementary regions, or require chimeric primers or chemically modified primers, resulting in higher costs and/or more complicated primer design. Some especially convenient universal seamless LIC protocols [7, 8, 15] involve the generation of single-stranded ends through exonuclease treatment without a defined stop position (sequence- and ligation-independent cloning, SLIC). The single-stranded ends can therefore exceed the length that would be required for intermolecular insert-to-plasmid annealing. Consequently, the circular plasmids resulting from insert-to-plasmid annealing can contain single-stranded gaps of diverse lengths (Fig 1C). In the context of molecular cloning, it is typically assumed that such gaps are well tolerated in *E*. *coli*, although the maximum gap-length that is efficiently repaired by the bacteria is not known [7, 15]. In contrast, early research on the substrate specificity of recBC deoxyribonuclease, a bacterial nuclease, indicated that single-stranded gaps in circular double-stranded DNA molecules can render the DNA vulnerable towards the nuclease [16].

The problem of single-stranded gaps has been solved by the Gibson assembly (Fig 1D), a method that can be used to assemble multiple very large DNA fragments [9]. In the Gibson assembly, single-stranded 3’-overhangs are produced using T5 exonuclease. After insert-to-plasmid annealing at cohesive complementary single-stranded ends, these overhangs serve as primers for DNA polymerase extension. In this way, Phusion DNA polymerase fills in unwanted single-stranded gaps. The remaining nicks are then sealed by *Taq* DNA ligase. The protocol consists of a convenient isothermal incubation step, with all three enzymes in one mixture. The only disadvantage of the method is that the *Taq* DNA ligase in the mixture makes the assembly mix rather costly.

Currently thriving genome editing methods such as the CRISPR-Cas9 technology [10] require the construction of entry clones, with the gene of interest flanked by long homology regions for subsequent introduction into a genome. Furthermore, there is a rising need for the co-expression of multiprotein complexes for structural biology [17], which is also typically achieved by methods requiring the production of entry plasmids. Although synthetic genes are often ordered in the context of ready-for-expression plasmids these days, applications such as structural biology or functional studies often require many iterative modifications to the original constructs. For such purposes, there is a need for simple, robust seamless cloning methods that are affordable and compatible with automation (involving only liquid handling and thermal cycling steps).

We here examined the importance of single-stranded cohesive DNA ends in co-transformation cloning and the influence of single-stranded gaps in circular double-stranded plasmids on SLIC cloning efficiency. Our data indicate that co-transformation cloning primarily works independently from single-stranded termini, and that post-PCR treatment of insert and plasmid with an exonuclease is required to obtain a sufficiently large subpopulation of DNA fragments with cohesive ends to significantly increase cloning efficiency via a SLIC-mechanism. Our data furthermore suggest that single-stranded gaps in circular plasmids have a strong negative effect on transformation efficiency. Based on this information, we developed a modified Gibson assembly protocol without ligase, which allows insert-plasmid assembly at the high efficiency of the Gibson assembly reaction, at a low price comparable to SLIC. This method meets all the prerequisites for the applications described above.

## Results and Discussion

### PCR-linearized plasmids and restriction enzyme digested plasmids are equally well suited for co-transformation cloning

The co-transformation of *E*. *coli* with a linearized plasmid and an insert PCR product containing suitable homology regions is extremely convenient, but the low efficiency of this method can lead to experiment failure. Improved efficiency would be highly desirable.

Co-transformation cloning (Fig 1A) has successfully been performed using either PCR-linearized [1, 4, 18, 19] or restriction enzyme digested/linearized [2, 20] plasmids. Long single-stranded termini in PCR-linearized plasmids, resulting from incomplete primer extension [21] can promote insert-plasmid assembly via an LIC mechanism [7, 22].

To test whether PCR-linearized plasmids boost insert-plasmid assembly in our standard co-transformation cloning protocol, we in parallel performed experiments with two different plasmid preparations. The NdeI / BamHI digested plasmid contained the same sequences at its terminal homology regions as the PCR-linearized plasmid. The short (2 nts and 4 nts) overhangs produced by the restriction enzymes were negligible for direct annealing, which for LIC requires a minimum of 10–12 nucleotides [23].

**Fig 2:**
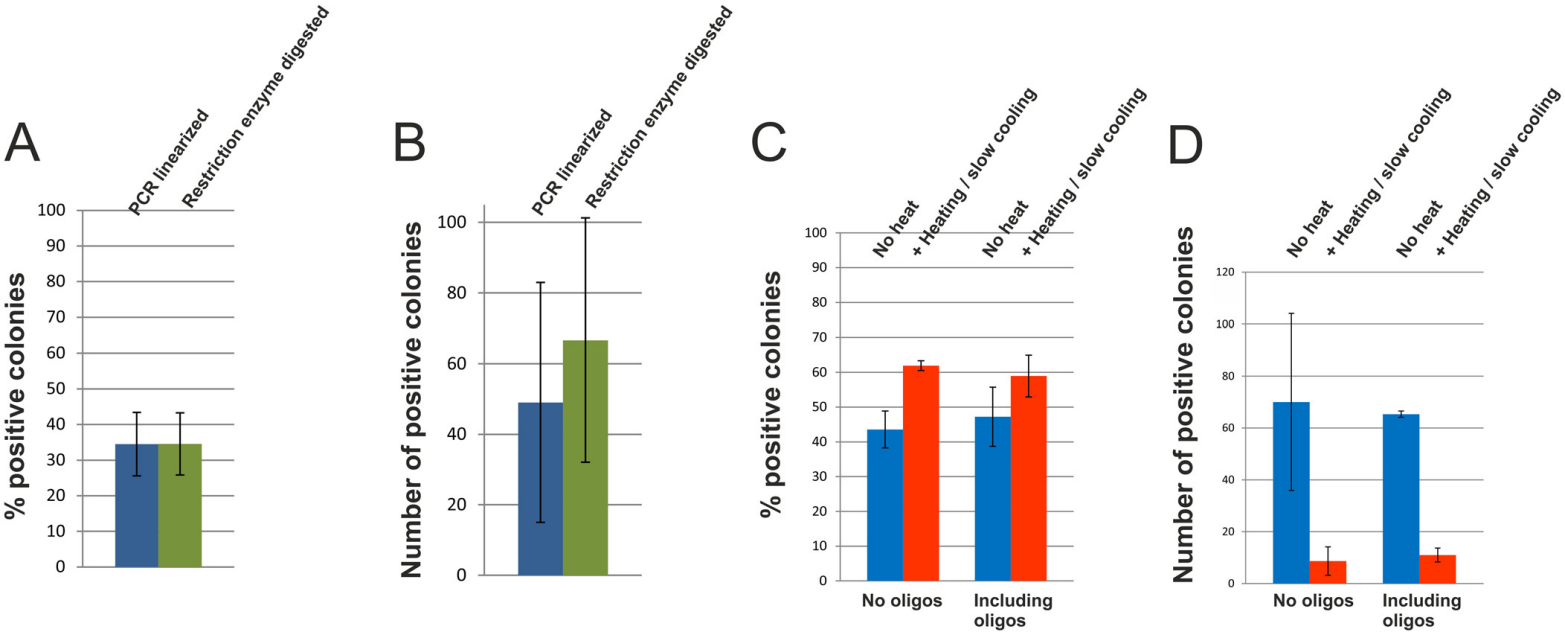
Critical factors for co-transformation cloning. A and B) *E*. *coli* cells were co-transformed with the lacZ insert PCR fragment and either PCR-linearized plasmid or restriction-enzyme digested/linearized plasmid. A) Data points represent the percentage of positive (blue) colonies averaged over three experiments ±SD. B) Data points represent the number of positive (blue) colonies averaged over three experiments ±SD. C and D) *E*. *coli* cells were co-transformed with the lacZ insert PCR fragment and PCR-linearized plasmid. The same experiment was also performed in the presence of a 10-fold molar excess over insert of each of two 15 nt long oligonucleotides comprising the sequence of the primer homology overhangs. All experiments were performed with and without a heat-denaturation / slow cooling step. C) Data points represent the percentage of positive (blue) colonies averaged over three experiments ±SD. D) Data points represent the number of positive (blue) colonies averaged over three experiments ±SD.

The percentage of resulting positive colonies was nearly identical for both plasmid preparations, and the total number of positive colonies was roughly in the same range, indicating that *in vitro* annealing, as in ligation-independent cloning, does not significantly contribute to insert-plasmid assembly in our standard co-transformation cloning protocol (Fig 2A and 2B). This indicates that the use of PCR-linearized plasmids is not a promising strategy for achieving improved results with this protocol.

It is interesting to note in this context that the mechanism of the very popular QuikChange mutagenesis protocol has very recently been revised [24]: For a long time, it was assumed that PCR-extension products contain cohesive 5’ overhangs that anneal *in vitro*, as in LIC. The revised mechanism [24] suggests the formation of fully double-stranded DNA products that circularize via homologous recombination *in vivo*, independent of single-stranded cohesive overhangs, as in co-transformation cloning.

### DNA denaturation-renaturation can strongly reduce transformation efficiency in co-transformation cloning

**Fig 3:**
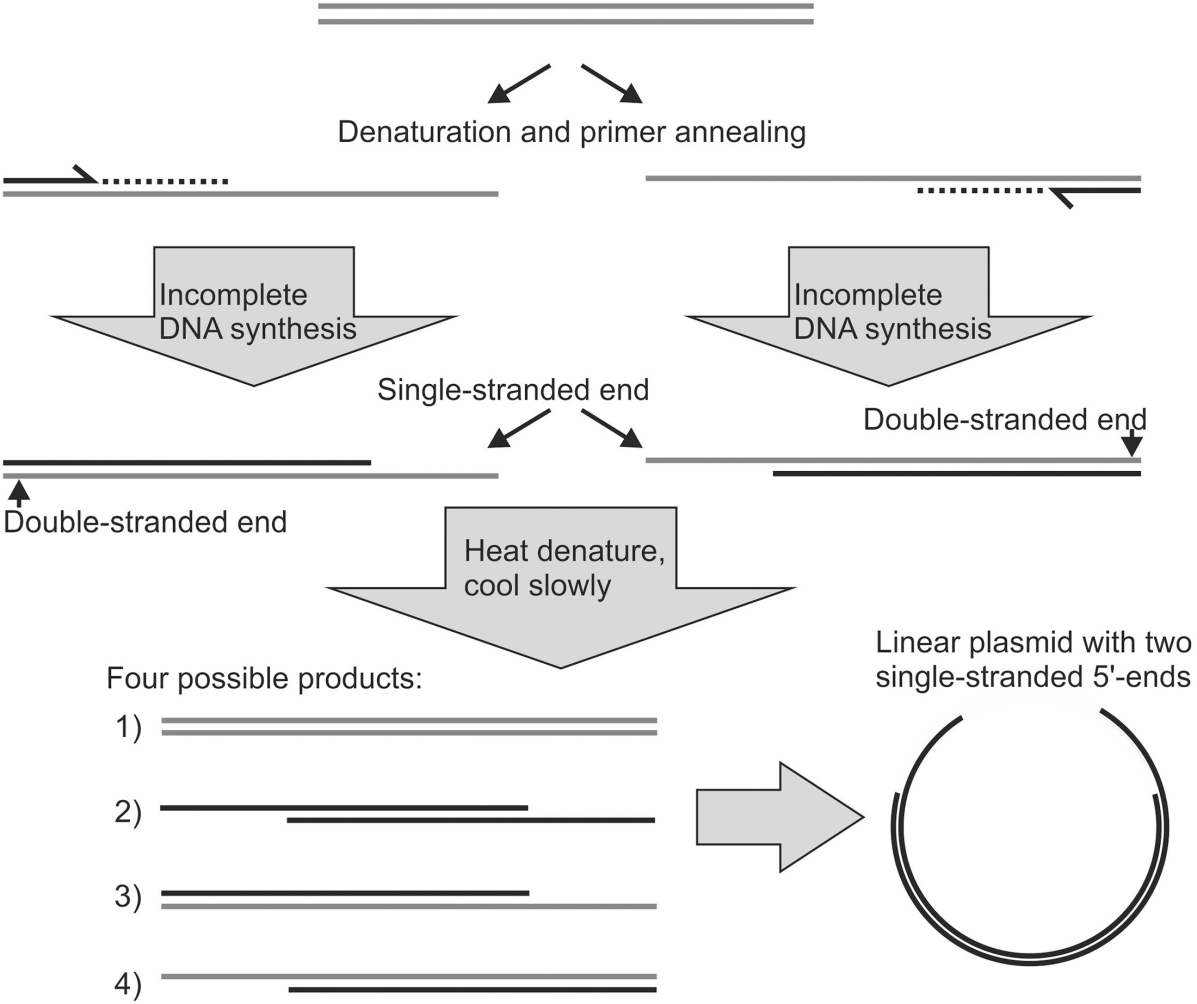
Producing two single-stranded ends by incomplete PCR. If primer extension in late PCR cycles is incomplete in a subset of PCR products, double-stranded DNA molecules with a single-stranded end may form. To produce double-stranded DNA molecules with two single-stranded ends, the PCR mixtures have to be denatured after the final polymerase extension step to allow new strand combinations upon renaturation. The concept shown here for the plasmid also applies to the insert. By the same mechanism, double-stranded DNA molecules containing two single-stranded ends could potentially form during PCR cycling if critical reagents are used up prior to the last PCR cycle(s), so that no DNA synthesis takes place in the last cycle(s).

We have not observed a significant increase in co-transformation cloning efficiency when using plasmids that were PCR-linearized using Phusion polymerase, compared to when restriction enzyme digested plasmids were used. However, a positive influence of single-stranded ends from incomplete PCR on cloning efficiency has been described when *Taq* DNA polymerase was used [7], which is known to produce incompletely extended strands [21] but may have other disadvantages, such as lower fidelity. In SLIC using PCR products containing single-stranded ends from incomplete PCR extension, a final denaturation / renaturation step without polymerase extension promotes the formation of double-stranded DNA molecules containing two single-stranded ends [7] (Fig 3). This final denaturation / renaturation step is not part of typical co-transformation protocols.

We were interested whether this approach would allow us to improve the efficiency of our co-transformation cloning experiments. To our surprise, heating, followed by slow cooling of insert-plasmid mixtures, resulted in a drastic drop in the number of resulting colonies (Fig 2D). This negative effect of heating can lead to failure of co-transformation cloning experiments, for example if heating is used for the inactivation of restriction enzymes, e.g. *DpnI*. The reason why the denaturation procedure had such a negative effect is unclear.

Linear DNA molecules are quickly degraded in *E*. *coli* [25]. Once linear insert and plasmid molecules are inside the cells (without *in vitro* generated single-stranded ends that allow annealing), there may be a struggle between DNA recombination (forming stable circular dsDNA) and degradation of the linear DNA molecules. This would also explain the low efficiency generally observed in co-transformation cloning, compared to other cloning methods such as ligation-independent cloning. We speculate that the denaturation–renaturation step could possibly lead to structural perturbations, such as the formation of hairpin loops, that render the already vulnerable linear DNA [25] molecules even more susceptible to degradation by *E*. *coli* nucleases. An increased vulnerability to nucleases caused by structural perturbations would also provide a speculative explanation for the long-standing puzzle why electroporation does not work for co-transformation cloning [2, 11], unless a special *E*. *coli* strain is used [11], which interestingly has inactivated RecB and RecC, parts of a nuclease complex that degrades linear DNA in *E*. *coli* [25]. Electroporation might lead to local heat generation and/or disruption of electrostatic interactions, which could perturb the DNA structure in similar ways as *in vitro* heat denaturation / renaturation. Electroporation is performed in the absence of salt. Low salt conditions favor strand separation: In the absence of counterions, there is significant electrostatic repulsion between negatively charged phosphates in the backbones of the two DNA strands [26]. In agreement with this speculative explanation, JC8679, an *E*. *coli* strain with an inactivated RecBCD enzyme [11], does work for co-transformation cloning using electroporation [11], but is not RecA-deficient, which could lead to unwanted recombination in the plasmids [27].

### Oligonucleotides do not inhibit recombination

The primers used for insert amplification and plasmid linearization contain the homology/overlap regions at their 5’-ends and hence could potentially compete with insert-to-plasmid annealing or recombination, if unpurified PCR products are used for co-transformation cloning. To investigate the effect of competitive oligonucleotides in more detail, we designed two 15 nt long oligonucleotides that comprised the exact sequences of the homology/overlap regions at which inserts and plasmids recombine. Because these oligonucleotides could also compete with the insert for annealing to the plasmid via *in vitro* generated single-strands (iPCR), we performed co-transformation cloning experiments in the presence of a 10-fold molar excess over insert of each oligonucleotide both, with and without heat denaturation–renaturation. The number of positive colonies was not significantly reduced compared to control experiments without oligonucleotides, neither without nor with heating-cooling. The results are shown in Fig 2C and 2D. The percentage of positive colonies was nearly identical amongst non-heated experiments, and amongst the heated experiments. These data support a recombination process that is independent of *in vitro* annealing, and suggest that removal of excess PCR primers is not critical for successful co-transformation cloning.

### Single-stranded gaps in circular double-stranded plasmids drastically reduce cloning efficiency

While *in vitro* insert-to-plasmid annealing does not significantly contribute to co-transformation cloning, the cloning efficiency of the same insert and plasmid preparations can be drastically increased through the generation of single-stranded termini that do result in *in vitro* insert-to-plasmid annealing at complementary single-stranded regions.

**Fig 4:**
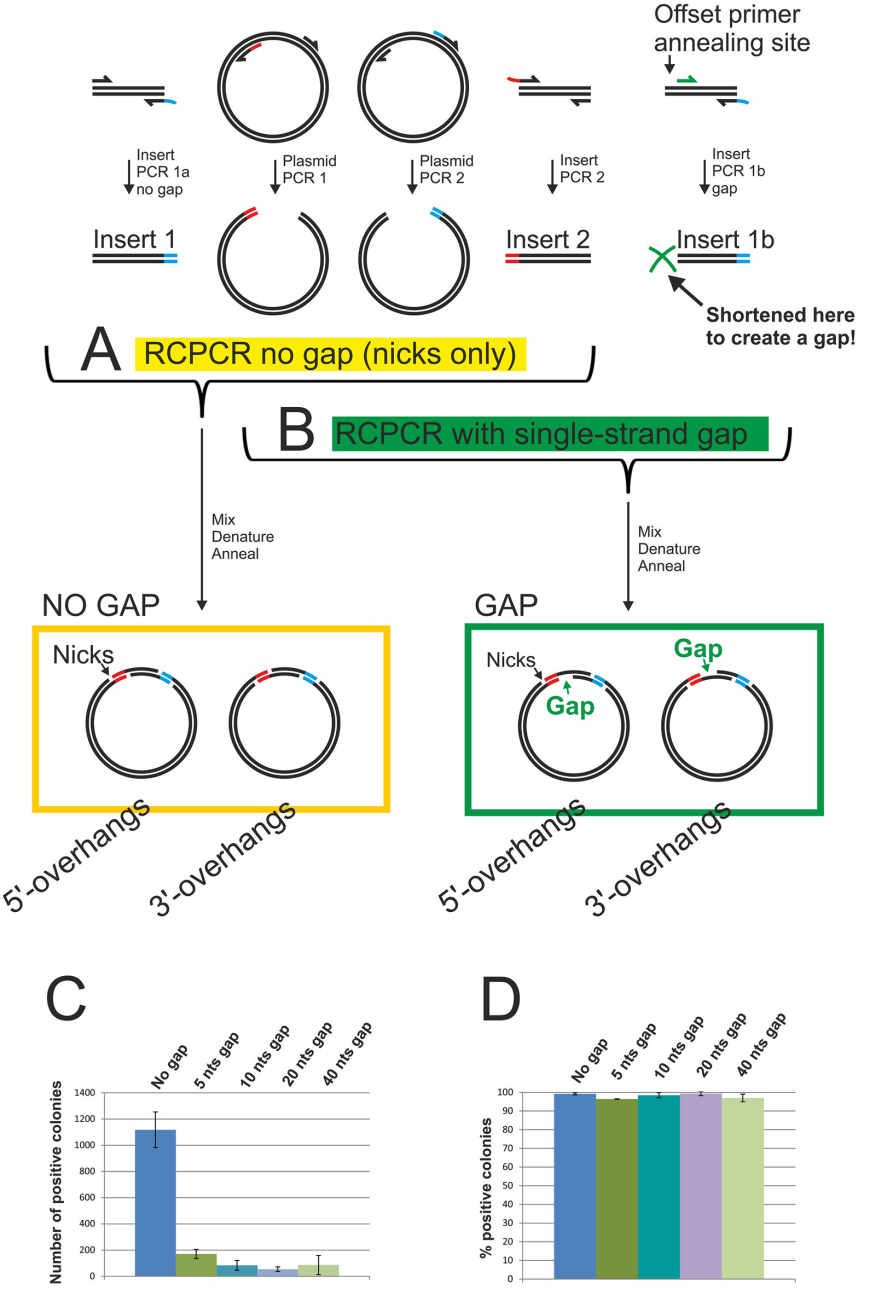
Single-stranded gaps in plasmids reduce cloning efficiency. A) Schematic overview of the concept of recombinant circle PCR (RCPCR)[28]. The insert and the plasmid are each amplified in two separate PCR reactions. One of the resulting insert and plasmid PCR products contain only homology region 1 (red). The other insert and plasmid PCR products contain only homology region 2 (blue). The four PCR products are mixed, heated and allowed to cool slowly, allowing new strand combinations. This results in the formation of double-stranded DNA molecules that can circularize through insert-to-plasmid annealing at single-stranded terminal complementary regions. B) A further downstream primer annealing site (green arrow) was chosen for PCR 1b. The resulting PCR 1 products lacking homology region 1 (red) were therefore shortened (green cross), and in combination with insert 2 and the two plasmid PCR products, after heating and slow cooling, formed a circular plasmid containing a single-stranded gap in addition to the nicks. The length of the gap depended on the (green) primer annealing site and for our experiments measured 5, 10, 20 or 40 nucleotides, respectively. C) Chemically competent *E*. *coli* cells were transformed with the plasmids described in (A) and (B). Plasmids with no gaps, or with 5, 10, 20 or 40 nucleotides long single-stranded gaps were tested. Data points represent the number of positive (blue) colonies averaged over three experiments ±SD. D) Percentage of positive (blue) colonies. Data points represent the percentage of positive colonies averaged over three experiments ±SD.

In sequence- and ligation-independent cloning (SLIC) methods involving single-stranded regions that are longer than needed for annealing, it was typically assumed that the single-stranded gaps resulting upon insert-to-plasmid annealing are efficiently repaired in *E*. *coli* [7, 15]. To test this, we used a recombinant circle PCR (RCPCR) [28] / enzyme-free cloning [29] approach (Fig 4A). Although this technique involves twice the number of primers and of PCR reactions compared to co-transformation cloning or typical LIC protocols, the method was ideally suited here because it allowed the creation of single-stranded gaps of precise length. The original RCPCR product contained nicks but no gaps. By choosing a different forward primer annealing region, we then shortened the insert PCR product lacking the first homology region by 5, 10, 20 or 40 basepairs. Hence, the circularized plasmids after the heating-cooling procedure not only contained nicks, but also a single-stranded gap (Fig 4B).

The efficiency at which the plasmids transformed *E*. *coli* cells dropped drastically when the RCPCR products contained a gap. The most significant drop in colony numbers was observed between RCPCR products with no gap and RCPCR products containing a gap 5 nucleotides in length. Longer gaps of 10 nts and 20 nts, respectively, resulted in a slight further decrease in cloning efficiency, while a 40 nts long gap did not appear to result in a further efficiency decrease. The presence or absence of a gap hence seems to be the most critical factor for high-efficiency LIC-based cloning, while gap length appears to play only a minor role. The results are shown in Fig 4C and 4D.

### Filling-in of single-stranded gaps restores cloning efficiency to a higher level in the Gibson assembly

**Fig 5:**
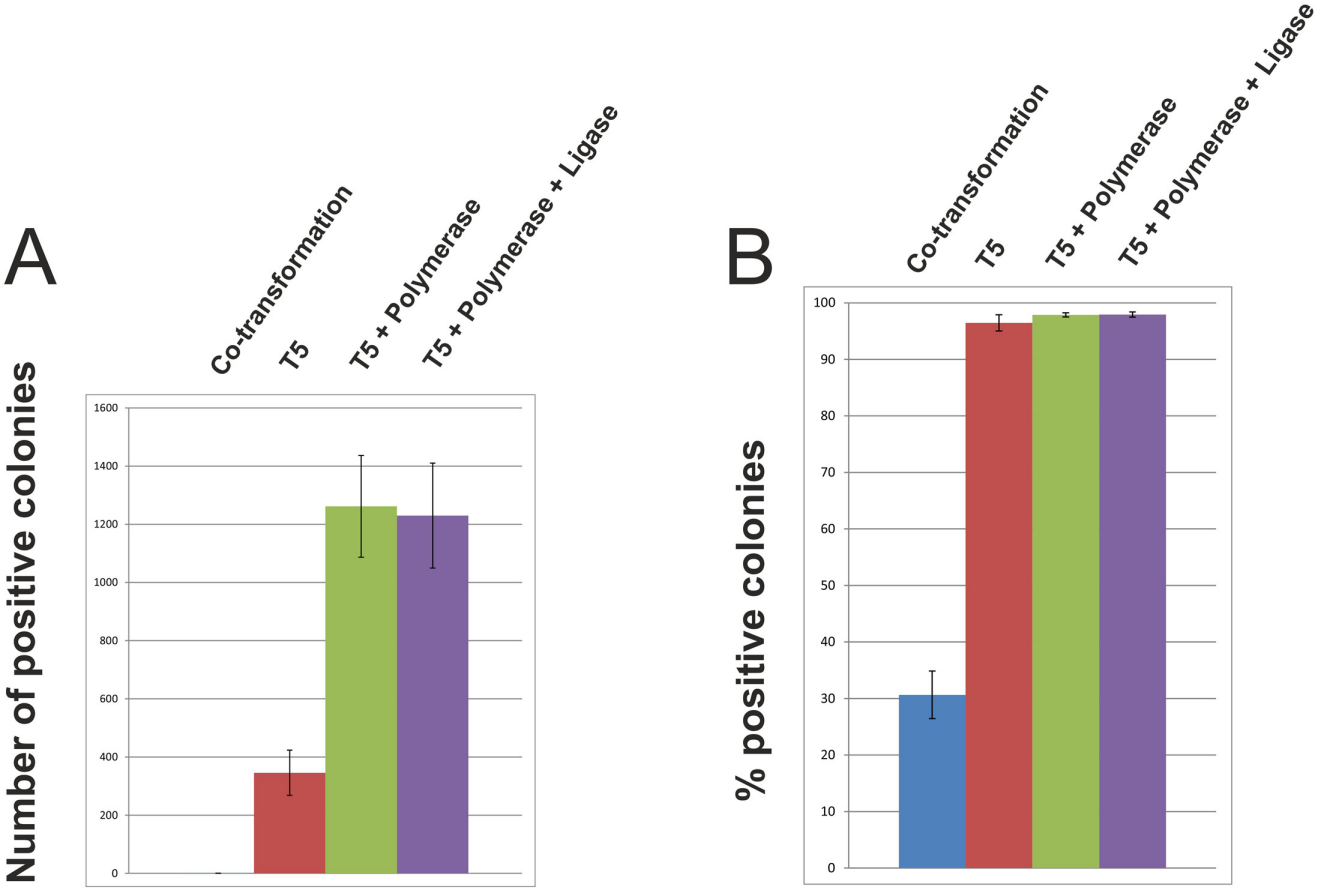
“Dissection” of the Gibson assembly into its component reactions. In the Gibson assembly, single-stranded 3’-overhangs are produced using T5 exonuclease. After insert-to-plasmid annealing at complementary single-stranded DNA ends, gaps are filled-in by Phusion DNA polymerase, and finally, *Taq* DNA ligase ligates the nicks. Here, we compared the efficiencies at which insert-plasmid mixtures transformed chemically competent *E*. *coli* cells. We used untreated insert-plasmid mixtures (co-transformation cloning), insert-plasmid mixtures treated with T5 exonuclease (sequence- and ligation-independent cloning), insert-plasmid mixtures treated with T5 exonuclease and Phusion DNA polymerase (sequence- and ligation-independent cloning plus gap filling), and insert-plasmid mixtures treated with T5 exonuclease, Phusion DNA polymerase and *Taq* DNA ligase (Gibson assembly). A) Data points represent the number of positive (blue) colonies averaged over three experiments ±SD. B) Data points represent the percentage of positive colonies averaged over three experiments ±SD.

The Gibson assembly (Fig 1D) [9] elegantly solves the problem of single-stranded gaps, because the latter are filled in by DNA polymerase, and the remaining nicks are then ligated by *Taq* DNA ligase. If single-stranded gaps reduce cloning efficiency, as we have shown in the RCPCR experiments, then filling in of gaps should boost cloning efficiency. To test this, we dissected the Gibson reaction into its components. We performed the Gibson assembly with all the components (exonuclease, polymerase and ligase), with exonuclease and polymerase only (filling of gaps but no ligation), with exonuclease only (single-stranded gaps) or without enzymatic treatment (co-transformation cloning). Treatment with T5 exonuclease already resulted in a more than 1000-fold increase in the number of positive colonies compared to co-transformation cloning, indicating that T5 exonuclease, at the temperature and in the buffer typically used for the Gibson assembly, is an excellent choice for sequence- and ligation-independent cloning. Filling-in of single-stranded gaps by Phusion DNA polymerase led to a further ~3.7-fold increase in cloning efficiency. Addition of *Taq* DNA ligase however did not result in a further increase in cloning efficiency. The results are shown in Fig 5. The ligation step is probably important for the assembly of (multiple) DNA molecules in the size range of hundreds of kilobases, for which the method was originally developed by Gibson et al. [9]. Our results however indicate that for simple insert-plasmid assemblies, the ligase can be omitted. *Taq* DNA ligase is the most costly component of the Gibson assembly. By omitting this enzyme, the price of the assembly mixture can be reduced significantly. Our experiments furthermore show that the cloning strategy can be used with overhangs that are short enough to be introduced to the inserts via PCR through non-annealing primer 5’-overhangs. Here, we used two overhangs 15 nucleotides in length, but the required length is likely to depend on the sequence and GC content at the desired cloning sites. The cloning strategy is an invaluable tool for the seamless construction of expression plasmids, independent of fixed cloning sequences such as unique restriction enzyme recognition sites.

## Materials and Methods

### Overall strategy

The lacZ α-peptide [30] ORF, under control of the *E*. *coli* tet promoter, was used as the insert, to allow easy identification of positive and background colonies by blue-white screening. The template used for insert amplification carried an Ampicillin resistance gene, while the target plasmid conferred resistance to Kanamycin, the insert template hence could not contribute to background colonies. Ten positive colonies from each type of experiment were sequenced to verify formation of the desired product. To allow direct comparison of different methods in parallel, preliminary experiments (not shown) where performed before each set of experiments to determine the amount of DNA that was required to allow reliable colony counting. Preliminary experiments (data not shown) furthermore showed that a large molar excess of insert over plasmid was favorable to achieve good cloning efficiency and a high percentage of positive clones. Therefore, depending on the methods that were compared, an 8–20-fold molar excess of insert was used (the amounts of DNA that were used are specified below for each experiment).

### Preparation of DNA samples

PCRs were performed using Finnzymes Phusion PCR Master Mix. Co-transformation cloning experiments: The primers Lin-1 (5’-CATATGGGGCCCCTGGAACAGAACTTCCAGGCC-3’) and Lin-2 (5’-GGATCCGAATTCGAGCTCCGTCGACAA-3’) were used for plasmid linearization. Homology regions are underlined. The primers lacZ-1-For (5’-CAGGGGCCCCATATGTTACAATTTCCATTCGCCATTCAGGCTGCGCA-3’) and lacZ-2-Rev (5’-CTCGAATTCGGATCCGTTTGACAGCTTATCATCGAATAG-3’) were used for insert amplification, using Novagen pETBlue-1 as the template. The PCR products were DpnI digested, spin column purified and eluted in 10 mM Tris pH 8.5. Plasmid linearization by digestion with restriction enzymes (NdeI / BamHI) was performed according to New England Biolabs guidelines.

### Co-transformation cloning

For co-transformation cloning, the linearized plasmid and the insert were directly added to 50 μl aliquots of chemically competent *E*. *coli* cells and incubated on ice for 15 minutes with occasional gentle shaking. Next, heat shock transformation was performed. Amount of DNA used for co-transformation cloning using PCR-linearized plasmid versus restriction enzyme digested plasmid: 85 ng plasmid and a 20-fold molar excess of insert. For co-transformation cloning using PCR-linearized plasmid with and without competing oligonucleotides and with and without heating—slow cooling, 170 ng plasmid and a 20-fold molar excess of insert were combined in a final volume of 6.5 μl in 10 mM Tris, pH 8.5, 10 mM MgCl_2_. In the experiments including competing oligonucleotides, the oligonucleotides (10-fold molar excess over insert) were incubated with PCR-linearized plasmid in 10 mM Tris, pH 8.5, 10 mM MgCl_2_ for 5 minutes prior to addition of the insert. Sequences of the oligonucleotides: Anti-F: 5’-CAGGGGCCCCATATG-3’ and Anti-R: 5’- CTCGAATTCGGATCC-3’. Aliquot 1 was heated to 95°C for 3 minutes (in the PCR machine with a hotlid) and then allowed to cool slowly to room temperature during 1 hour in a styrofoam rack. Aliquot 2 was not heated. The complete mixtures were used to transform 50 μl aliquots of chemically competent *E*. *coli* cells.

### Transformation

Novagen NovaBlue *E*. *coli* cells were used for all experiments. Genotype: *endA1 hsdR17* (r_K12_^−^ m_K12_^+^) *supE44 thi-1 recA1 gyrA96 relA1 lac* F’[*proA*^*+*^*B*^*+*^ *lacI*^*q*^*ZΔM15*::Tn*10*] (Tet^R^). For co-transformation cloning, NovaBlue Singles were used (transformation efficiency ≥ 1.5 x 10^8^ cfu/μg). For SLIC and the Gibson assembly, as well as for the RCPCR experiments, chemically competent NovaBlue cells were prepared according to the method described by Inoue et al. [31] (transformation efficiency ≥ 1 x 10^7^ cfu/μg). Heat-shock transformation was performed at 42°C for 45 seconds. After addition of 300 μl S.O.C. medium and incubation at 37°C on a shaker-incubator for 1 hour, the complete transformation mixtures were plated onto LB agar plates containing 50 μg/ml Kanamycin, 40 μg/ml IPTG and 100 μg/ml Bluo-gal or 40 μg/ml X-Gal.

### RCPCR

Insert amplification primers: PCR A: lacZ_no_H_For: 5’-TTACAATTTCCATTCGCCATTCAGGCTGCGCA-3’ and lacZ-2-Rev (see above). PCR B: lacZ_no_H_Rev: 5’-GTTTGACAGCTTATCATCGAATAG-3’ and lacZ-1-For (see above). Plasmid linearization primers: Linearization PCR C: Lin_no_H1: 5’-GAACAGAACTTCCAGGCCGCTGCTGTGATGATGATGATGATG-3’ and Lin-2 (see above). Linearization PCR D: Lin_no_H2: 5’-CTCCGTCGACAAGCTTGCGGCCGCACTCGAGCAC-3’ and Lin-1 (see above).

All PCR products were digested with DpnI, spin column purified and eluted in 10 mM Tris, pH 8.5. The two plasmid PCR products (45 ng each) and the two insert PCR products (10-fold molar excess) were combined in 10 mM Tris, pH 8.5, 15 mM MgCl_2_, 30 mM NaCl in a final volume of 7 μl per aliquot. The mixtures were heated to 95°C for 3 minutes (in the PCR machine with a hotlid) and then allowed to cool slowly to room temperature during 1 hour in a styrofoam rack. The complete mixtures were used to transform 100 μl aliquots of chemically competent *E*. *coli* cells. For RCPCR experiments with gaps, primer lacZ_no_H_For was replaced by: lacZ-5gapF (5 nt gap): 5’-ATTTCCATTCGCCATTCAGGCTGCGCAAC-3’, lacZ-10gapF (10 nt gap): 5’-CATTCGCCATTCAGGCTGCGCAACTG-3’, lacZ-20gapF (20 nt gap): 5’- TCAGGCTGCGCAACTGTTGGGAAG-3’ lacZ-40gapF (40 nt gap): 5’-GAAGGGCGATCGGTACGGGCCTC-3’.

### Gibson assembly

The same insert and PCR-linearized plasmid preparations as for co-transformation cloning were used. The 5x isothermal reaction buffer (5x IT buffer) consisted of 25% PEG 8000 (Sigma P5413), 500 mM Tris HCl pH 7.5, 50 mM MgCl_2_, 50 mM DTT, 1 mM of each dNTP (New England Biolabs N0447S) and 5 mM NAD^+^ (New England Biolabs B9007S) [9]. An insert-plasmid mastermix was prepared. Each Gibson assembly reaction consisted of 2.7 μl 5x IT buffer, 2 μl insert-plasmid mastermix (containing 75 ng plasmid and an 8-fold molar excess of insert), 5.3 μl 1:1000 diluted T5 exonuclease (New England Biolabs M0363S, 10’000 U/ml), 1.6 μl of 1:10 diluted Phusion HF DNA polymerase (NEB M0530L, 2’000 U/ml), 1.3 μl Taq DNA ligase (NEB M0208L, 40’000 U/ml, undiluted) and H_2_0 to a final volume of 13.5 μl. In the reactions without *Taq* DNA ligase and in the reactions without *Taq* DNA ligase and without Phusion DNA polymerase, these enzymes were left out and the final volume was adjusted to 13.5 μl with nuclease-free water. The reactions were incubated in the PCR machine at 50°C for one hour with the hotlid at 80°C. After cooling down to 4°C, 1/20 (3.75 ng plasmid and an 8-fold molar excess of insert) of each of the mixtures was transferred to 100 μl aliquots of chemically competent *E*. *coli* cells and incubated on ice for 15 minutes, followed by heat shock transformation. 300 μl of S.O.C. medium were added to the cells. After recovery for one hour at 37°C, 650 rpm, the complete transformation mixtures were plated out. In the absence of enzymatic treatment (co-transformation cloning), transformation of only 1/20 of the amount of DNA was insufficient to obtain transformants, therefore, all the DNA was transformed and the result was divided by 20 for the comparison.
